# Adverse childhood experiences and psychological distress among higher education students in Southeast Nigeria: an institutional-based cross-sectional study

**DOI:** 10.1186/s13690-021-00587-3

**Published:** 2021-04-29

**Authors:** Olaoluwa Samson Agbaje, Chinwe Patience Nnaji, Evelyn Nwanabe Nwagu, Cylia Nkechi Iweama, Prince Christian Ifeanachor Umoke, Lawretta Eyuche Ozoemena, Charles Chike Abba

**Affiliations:** Department of Human Kinetics and Health Education, Faculty of Education, University of Nigeria, Nsukka, Enugu State Nigeria

**Keywords:** Adverse childhood experiences, Psychological distress, Mental health, Young adults, Higher education, Nigeria

## Abstract

**Background:**

Exposure to adverse childhood experiences (ACEs) constitutes public health problems linked to adverse mental outcomes such as psychological distress during adulthood. This study examines the prevalence of ACEs and psychological distress and explores the association between ACEs and psychological distress and demographic factors among young adults.

**Methods:**

We conducted a cross-sectional study of 330 students from May 2018 to July 2018. The participants completed the Adverse Childhood Experiences International Questionnaire (ACE-IQ), Kessler Psychological Distress Scale (K10), and the sociodemographic profile scale. We used descriptive statistics to describe the prevalence of ACEs and psychological distress in our sample. After adjusting for the demographic covariates, ACEs’ association with psychological distress was determined using binary and multivariate logistic regressions.

**Results:**

A total of 203 students with a mean age of 20.76 ± 2.73 years completed the study. The total mean ACE score was 4.58 ± 1.59, and the total mean psychological distress score was 20.76 ± 6.31. Most of the participants (86.7%) experienced ACEs, 14.8% reported experiencing one ACE, 30.5% reported experiencing 2–3 ACEs, and 41.3% reported experiencing 4+ ACEs. Further, about 85% of the youth have experienced at least one form of sexual abuse during childhood, and females reported a higher number of ACEs than males. Sexual abuse (OR = 2.36; 95% CI: 2.36, 7.65), physical neglect (OR = 2.87; 95% CI: 1.57, 5.31), overall ACE exposure (OR = 6.66; 95% CI: 2.41, 18.42), having 1 ACE (OR = 4.40; 95% CI: 1.32, 14.70), having 2–3 ACEs (OR = 4.13; 95% CI: 1.39, 12.29), and having 4+ (OR = 11.67; 95% CI: 3.95, 34.45) were significantly associated with psychological distress.

**Conclusions:**

ACEs are prevalent among young adults and are associated with psychological distress in adulthood. Furthermore, parental factors are associated with ACEs and psychological distress. Thus, implementation of school, community-and facility-based routine mental health screening programs is essential for prompt identification, prevention, and treatment of youth with childhood adversities and poor mental health outcomes.

**Supplementary Information:**

The online version contains supplementary material available at 10.1186/s13690-021-00587-3.

## Background

Mental health is a significant dimension of the overall well-being of young people. It also implies the capacity to perform in terms of their thoughts, feelings, and behaviors successfully. Having these capacities helps young people become productive, enjoy fulfillment in relationships with others, and effectively adapt, change, and cope with life challenges [1]. Young people or youths are vulnerable to mental health problems in diverse communities. Mental health problems affect about 10–20% of children and adolescents worldwide [1]. Most mental disorders begin during the active phase of life-12-24 years of age [2]. Evidence has shown a link between exposure to adverse or traumatic experiences during childhood and the development of adverse health outcomes such as mental and physical health problems in later life [3–5]. The ACEs refer to “harmful experiences that occur in early childhood and have a strong potential to traumatically affect the health of the individuals experiencing them” [6]. Although ACEs’ definition has evolved and varied in diverse contexts since its first use in the literature [5, 7], there are recent debates on what constitutes ACEs and the need for conceptual clarification [8, 9].

A study [8] advocated an inclusive definition that considers the effects of the socioeconomic determinants of health on ACEs. According to the authors, this approach may provide significant insights into the importance of socioeconomic conditions in children’s traumatic and abusive experiences. However, there is a consensus on experiences or events that constitute ACEs [5–8]. ACEs encompass children’s traumatic and abusive experiences caused by parents, guardians, caregivers, teachers, or other adults either directly or indirectly [5–9]. The adverse events include exposures to childhood psychological, physical, or sexual abuse; domestic abuse and violence against the child’s mother; living with household members who had substance misuse disorders, were mentally ill or suicidal or had been jailed in the past and loss of a parent through divorce, separation or death [6, 7, 10–12].

ACEs are associated with poor health outcomes such as cancer, heart disease, emphysema, premature mortality, stroke, depression, substance use, obesity, unemployment, and persistent violence perpetration by victims [13–20]. Research suggests a link between ACEs exposure and psychological outcomes in children and young people [1, 4, 7]. One of such psychological consequences is mental distress. The prevalence of psychological distress (PD) has been reported in young people [13, 16]. Indicators of PD include poor mood, anxiety, depression, and stress. PD has been shown to the harbinger of mental illness and other worse outcomes in children and adolescents for several years and manifests in adult life [21–23].

Studies have shown that psychological distress, like other mental health problems, is associated with several factors at different social-ecological levels [2, 24]. Such factors exist at the individual, family, school, and community levels. The individual factors include low self-esteem, depression, anxiety, aggressiveness, sadness, loneliness, while the family-related factors include domestic violence, little emotional support, difficulties within family relationships, and lack of communication. The school-level factors include low performance/academic failure, drop-out, and bullying. The community-level factors include exposure to violence and aggression, sexual abuse, social problems and rules violation, community disorganization, legal issues, and poverty.

Furthermore, studies suggest that there is a high prevalence of childhood adversities among Nigerian children and adolescents. For example, Oladeji, Makanjuola, and Gureje [25] reported that almost half and 31.2% of their sample had experienced one adversity and two or more adversities. The results of the Nigeria Violence Against Children Survey (VACS) conducted in 2014 showed that before the age of 18 years, 6 in 10 female and male children had experienced at least one form of violence in childhood. Furthermore, approximately 1 in 6 adolescents aged 13 to 17 years had experienced two or more types (sexual abuse, emotional violence, physical violence, or physical and psychological violence) in the past 12 months [26]. The high prevalence of childhood adversities calls for valid data on young Nigerian adults’ mental health status.

Further, evidence suggests that the youthful stage is when mental disorders are often detected for the first time [2]. The availability of empirical data on childhood adversities could help formulate appropriate mental health policies and interventions for the Nigerian youth. However, there is limited empirical evidence on young people’s mental health status from low-and-middle-income countries (LMICs), including Nigeria [2, 27].

Research evidence shows that disparities exist in the prevalence of psychological distress among young people [28–32]. In this study, we hypothesized that young adults who experienced ACEs and lack certain protective factors in their lives would be more likely to experience psychological distress. This paper model the linkage between ACEs and later poor psychological outcomes (i.e., PD) to test whether parental characteristics such as education and income are associated with ACEs and PD. This hypothesis is supported by studies that have shown that parents or familial factors potentially disrupted the association between ACEs and poor mental health outcomes [33, 34]. This, we think, will add to the available data on ACEs and mental health outcomes and may enhance the implementation of appropriate interventions.

Thus, the present study sought to add to the available evidence on the association between ACEs and psychological distress among young adults, especially from developing countries. This study had two goals: (i) determine the prevalence of ACEs and psychological distress among Nigerian higher education students; (ii) determine the association between ACEs and later psychological distress. Although studies [35, 36] showed that there are protective factors (parental relationship/bonding, peer influence, and school connectedness) that mediate the association between ACEs and poor psychological outcomes in the family, neighborhood, and school, we examined only the family factors [37], the present study examined the association between young adults’ ACEs, psychological distress and some demographic factors. Understanding the links between ACEs and psychological distress and associated demographic factors in Nigerian youth may provide baseline data for measuring the impact of ACEs across populations [14, 21].

## Methods

### Study design, setting, and population

Nsukka Local Government Area (LGA) is in Enugu State, South-East Nigeria. It has 1810 km^2^ and a population of 309,633 at the 2006 census [38]. Nsukka LGA has a projected population of 417,700 in 2016, according to the National Bureau of Statistics [39]. The Local Government Headquarters is in the hilly and green sites, which Nsukka is known for close to colonial quarters of the pre-Independence years. Nsukka LGA has a few higher education institutions. These include the University of Nigeria, Nsukka (Nsukka Campus), the College of Education, Nsukka, and the School of Public Health Technology, Nsukka. Also, Nsukka LGA is an academic hub, and many young people seek academic pursuits in the town. A high proportion of these youth might have experienced adversities while growing up [26]. The present study is a cross-sectional institutional study conducted on 330 students of two tertiary institutions admitted to the Bachelor of Science (B.Sc./B.Ed.) Degree in Education and a Diploma in Community Health and Public Health Nursing at the University of Nigeria (Nsukka Campus) and School of Public Health Technology, Nsukka, respectively. We excluded the College of Education, Nsukka, from the study because approval to carry out the study in the institution was not given, and the data on the student population was not acquired.

### Sample size and determination procedure

The sample size was determined using the single population proportion formula [40, 41], considering the following assumptions: prevalence (*P*) of any form of adversities in Nigerian children and adolescents (31.2%) [25], a margin of error (*W*) = 5%, Z = 1.96 at 95% confidence interval. Thus, the calculated sample size was 330. Furthermore, we estimated the statistical power using the G*Power 3.1.9.4 software version [42]. We used the post hoc power analysis for the logistic regression procedure. We used the traditional value of α = 0.05 recommended by Hickey et al. [43], software version developed by the large sample approximations proposed by Demidenko [33] with variance correction. The distribution of the predictor *X*, the “X distribution,” and its parameters were specified. We specified the X distribution as normal and its parameters, X parm *μ*, and X parm σ as 0 and 1, respectively. Furthermore, we specified the number of “tail(s)” of the test as two, the *P*_1_ and *P*_2_ as 0.30 and 0.50 respectively, while the odds ratio was 2.33333. The “*R*^2^ and other X” was specified as .1, and the total sample was 330. The post hoc statistical power analysis results showed that the critical z = 1.95996, corresponding to α = *β* = 0.9999 (Additional file 1). The results indicate that the minimum sample size of 330 was enough for the study. A multistage sampling technique was used to select the participants. The participants who had severe psychological distress were referred to clinical psychologists and mental health experts. The Institutional Review Boards (IRB) at the Faculty of Education, University of Nigeria, Nsukka, reviewed and approved the study. Participants provided verbal informed consent before completing the surveys. The study was conducted from May to July 2018.

### Participant recruitment and enrollment

The students’ recruitment occurred through person-to-person interviews and interactions with the course representatives (Class representatives). To be eligible for the study, participants had to report having one ACE before gaining admission into a higher institution [26], experienced psychological distress and attained ≥18 years. We excluded respondents with illness from the study. These eligibility criteria were selected based on the objectives of the study. After providing verbal consent, the participants completed the surveys administered through a conducive classroom environment. The data were collected using two health educators (the health educators possessed B.Sc.) who had previous experience in data collection procedures. The data collection process was supervised by two experts who had a master’s degree and Ph.D. in Public Health Education and previous experience in research supervision.

### Data quality assurance

We conducted a pre-test on 30 individuals to assure the reliability and validity of the questionnaires. A pilot study was conducted before actual data collection for cultural appropriateness and cultural relevance, as recommended by Beals et al. [44]. The pilot study comprised 13 males and 17 females. The students were asked to comment more generally about what issues they experienced while responding to the items in the Adverse Childhood Experiences-International Questionnaire (ACE-IQ) and K10. In the ACE-IQ, we removed the questions that elicit information on cultural/ethnic group background, civic status, work status, and marital status because a significant proportion of the students was not legally married, either cohabitating or working. Also, many of the respondents were Igbos (a dominant ethnic group/tribe in Nigeria). The students reported no issues with the slightly modified ACE-IQ. We trained the data collectors and supervisors for 1 day on the data collection principles.

### Measures

#### Adverse childhood experiences

We assessed ACEs using the Adverse Childhood Experiences International Questionnaire [11, 12, 45, 46]. The ACE-IQ is an 11-item tool that has been used extensively to measure the occurrence of adverse events before age 18. The ACE-IQ measures the following adverse events: physical abuse; being touched sexually; attempted to be made to touch someone sexually; being forced to have sex; psychological or emotional abuse; living with an adult who was depressed, mentally ill, or suicidal; living with anyone who was a problem drinker or alcoholic; living with a drug user or abuser; living with someone who was incarcerated or jailed; having parents who were separated or divorced; and living in a home where adults or parents physically harmed each other [11, 45]. Examples of questions in the ACE-IQ included ‘While you were growing up, during your first 18 years of life: was anyone in your household depressed or mentally ill?’ and ‘During the first 18 years of your life how often did a parent, guardian or household member punch, kick or beat you?’ We structured ACE questions in line with the CDC’s short ACE tool. All ACE items were dichotomized to show if the respondents had ever experienced adverse events before age 18. ACE items such as physical abuse, sexual abuse, and adults in the household treated each other violently are assigned a 4-point Likert scale: *Always* = 1, *Most of the time* = 2, *Sometimes* = 3, *Rarely* = 4, and *Never* = 5. The participants’ ACEs were defined if they answered affirmative (whether with once, a few times, or many times). This counts as a “Yes,” and so was coded as *1*. However, if the respondents answered “Never,” it was coded as *0*.

Furthermore, some ACE questions such as “*did you live someone who is mentally ill/depressed/suicidal, a problem drinker or was jailed*” are assigned a response option of “Yes” or “No.” If the respondents answered ‘Yes’ to any item in this category, it was coded as *1*, and if they answered ‘No,’ it was coded as *0*. Consistent with a previous study [30], we did not collapse the items that measured sexual abuse aspects into a single measure. They were examined independently. Consequently, we examined eleven ACE measures in this study. ACEs scores were generated using category count and a summary count to show the range of exposures students reported (no exposures = 0; exposed to all forms of adversity =11). The Cronbach’s alpha for the ACE-IQ questionnaire in the current study was 0.87 (Additional file 2).

#### Psychological distress

The prevalence of PD was assessed using the Ten-item Kessler Psychological Distress Scale (K10). One of the most widely used tools for either screening or establishing the severity of non-specific psychological distress is the ten-item Kessler Psychological Distress Scale (K10) developed by Kessler et al. [29]. The K10 was used to measure psychological distress. The scale comprises ten questions that ask about distress experiences using a 30-day reference period. Each item ranges in severity from ‘none of the time,’ to ‘all of the time,’ on a scale from 1 to 5, with higher scores indicating a higher level of psychological distress [24]. Respondents are asked to describe how often they felt nervous, hopeless, restless, or fidgety, so sad that nothing could cheer them up, that everything was an effort, and worthless [46]. The Cronbach’s alpha for the K10 questionnaire in the current study was 0.92 (See Additional file 3).

#### Potential confounders

Also, the selection of potential confounders or covariates was informed by previous studies [47, 48]. Individual attributes examined explicitly in the models included age, gender, academic level, and birth order. We assessed these characteristics by a standardized form designed by the researchers. The variables were structured as follows: age was measured as a continuous variable. However, age was further categorized into three groups: 18–20 years (1), 21–24 years (2), and 25 years and above (3) for ease of identifying a group with more exposure to ACEs. For instance, gender (male or female), birth order, first child (1), second child (2), third child (3) ≥ fourth child (4) and the last child (5). According to the National University Commission (NUC), the year of study or academic level was categorized into four levels as required by the first-degree program in Nigeria.

### Data processing and statistical analysis

After primary data screening and cleaning were performed, we analyzed data to describe the students’ demographic characteristics, ACEs, and PD. Only 203 participants completed the survey with full information (response rate of 61.5%). Results of the continuous variables were expressed as means ± standard deviation (SD). Frequencies (percentages) were used to describe the sample characteristics, and categorical variables ACEs exposure (Yes vs. No), and psychological distress (presence/yes vs. absence/no). The normality of the continuous data was examined using the Kolmogorov-Smirnov test, and data distribution did not fulfill the criteria for normality (Table 2).

Consistent with previous studies [30, 45, 49], an ACE count was used to classify participants into four ACE groups. We computed a sum of scores for psychological distress experience using the K10 (the K10 scores range from 10 to 50), and as recommended, we dichotomized the scores. A score below 20 (< 20) represented the absence of PD (i.e., *No*), and a score of 20 or higher (≥ 20) implied the experience of PD (i.e., *Yes*) [50]. We created ACE scores according to the category count and a summary count to indicate the range of exposures students reported. Thus, the ACE score ranges from 0 to 11. A score of 0 implied no exposures, and a score of 11 implied exposures to all forms of adversities. The demographic variables were included in the analyses as covariates. Spearman-rank order correlation was used to determine the relationship between students’ ACEs Scores and K10 scores.

Cohen’s [51] guidelines were used for the interpretation of the correlation coefficient. We used the Chi-square test of independence to determine the students’ bivariate associations of ACEs, PD, and sociodemographic characteristics. We dichotomized ACEs into two categories of *exposure* (experience of ACEs/Yes) versus *no exposure* (never experienced ACEs/No). Also*,* binary logistic regression was used to examine the independent associations between ACEs and psychological distress in our sample. Multivariable logistic regression was used to model the association of ACEs and PD by demographic characteristics. We further created four ACE groups (0, 1, 2–3, and 4+) and *ACEs exposure* versus *no exposure* based on prior studies [6, 30, 31, 45]. We conducted bivariate and multivariable logistic regression analyses to identify factors associated with ACEs and psychological distress among the students. Variables with a *p*-value of < 0.05 in the bivariate analysis were further entered in the multivariable logistic regression model. The multivariable logistic regression analysis was conducted to adjust for potential confounding variables [52]. We calculated the crude odds ratios (ORs) and adjusted odds ratios (AORs) with their 95% confidence intervals (CIs). We used the ORs and AORs to evaluate the statistical strength of association with their 95% confidence intervals. We excluded questionnaires with items of more than 20% missing values from data analyses. Data analyses were performed using the IBM SPSS Statistics for Windows, version 24 (IBM Corp., Armonk, NY, USA). Model fitness was checked by using the Hosmer and Lemeshow goodness of fit test. Statistical significance was determined at *P* <  0.05.

## Results

### Participants’ characteristics

A total of 203 students with a mean age of 20.76 ± 2.73 years completed the study. More than half (55.2%) of the students were aged 18–20 years. Respondents were predominantly female (58.1%), and 33% were at the 300 level. About 27% of the sample were female first child, and more than 40% of them have a father or mother with secondary education. Also, 68.5 and 31.5% of the students came from the University of Nigeria, Nsukka, and the School of Public Health Technology, Nsukka (Table 1).

**Table 1:** Sociodemographic characteristics of the participants (*N* = 203), May to July 2018, Nsukka, Nigeria

Characteristics	*N* (%)
Age groups	
18–20 years	112 (55.2)
21–24 years	57 (28.1)
25 years & Above	34 (16.7)
Gender	
Male	83 (40.9)
Female	120 (59.1)
Academic Level/Year of Study	
100 Level	59 (29.1)
200 Level	50 (24.6)
300 Level	67 (33.0)
400 Level	27 (13.3)
*Parental Educational Level	
NFE	45 (22.2)
PRY EDU	37 (18.2)
SEC EDU	78 (38.4)
TER EDU	43 (21.2)
Birth Order	
First Child	54 (26.6)
2nd Child	53 (26.1)
3rd Child	16 (7.9)
≥ 4th Child	38 (18.7)
Last Child	42 (20.7)
Parental income^a^	
Low income	63 (37.7)
Middle	64 (38.3)
High income	40 (24.0)

*Note*. ^a^Any of the parents. *NFE* No Formal Education, *PRY EDU* Primary Education, *SEC EDU* Secondary Education, *TER EDU* Tertiary Education

### Association between ACE counts and psychological distress scores

The Spearman-rank order correlation between ACE scores and K10 scores showed a small positive relationship (*rho* = .46, *p* <  0.0001) (Table 2).

**Table 2:** Possible and observed ranges, mean, and standard deviations, correlation between adverse childhood experience scores and psychological distress scores May to July 2018, Nsukka, Nigeria

Continuous variables	Possible range	Observed range	Mean (SD)	*rho*	*p-value*	Kolmogorov-Smirnov test	
						*Statistic*	*p-value*
ACE-IQ score	0–11	0–10	4.58 (1.59)	.46**	< 0.0001	0.083	0.002
K 10 score	10–50	10–39	20.76 (6.31)			0.097	< 0.0001

** Correlation is significant at *p* <  0.0001

### Prevalence of ACEs

The total mean ACE score was 4.58 ± 1.59, and the total mean psychological distress score was 20.76 ± 6.31 (Table 4). Overall, most of the participants (86.7%) experienced ACEs. Besides, 14.8% of the participants reported experiencing one ACE, 30.5% reported experiencing 2–3 ACEs, and 41.3% reported experiencing 4+ ACEs (Table 3). Further, about 85% of the youth have experienced at least one form of sexual abuse during childhood, and females reported a higher number of ACEs than males (Table 3). Also, one-third (32.1%) of our sample reported experiencing one form of an adverse event due to household dysfunction (Table 3). Also, 70.9, 56.2, 51.7, and 25.6% of the participants, respectively, reported physical abuse, emotional abuse, physical abuse, emotional and physical neglect.

**Table 3:** Prevalence of each adverse experience in the sample and individuals with different adverse childhood experience counts, May to July 2018, Nsukka Nigeria

ACEs	All Sample^c^		
	*N* (%)	Mean	SD
*Did you experience any of the following events before the age of 18 years*?			
Household dysfunction			
Lived with someone who was mentally ill or suicidal	5 (2.5)	3.28	1.19
Lived with a problem drinker or alcoholic	18 (8.9)	2.86	1.10
Lived with someone who used illicit street drugs or abused prescription medications	3 (1.5)	2.62	1.06
Lived with someone who was jailed or imprisoned for a crime	8 (3.9)	3.21	1.15
Were your parents ever separated or divorced (parental separation)?	31 (15.3)	2.49	1.01
Physical abuse			
Did your parents or adults in your home ever			
Slap, hit, kick, punch, or beat each other up?	114 (56.2)	3.10	1.20
Hit, beat, kick, or physically hurt you in any way?	75 (36.9)	2.59	1.02
Emotional abuse			
Scream or Swear at you, insult you or put you down?	144 (70.9)	2.57	0.98
Sexual abuse			
Did anyone at least 5 years older than you (including adults)			
Ever touch you sexually?	73 (36.0)	2.47	0.99
Try to make you touch them sexually?	57 (28.1)	2.28	0.87
Force you to have sex?	42 (20.7)	2.17	0.75
Emotional neglect			
Did your parents/guardians understand your problems and worries?^a^	16 (7.9)	4.28	1.23
Did your parents/guardians really know what you were doing with your free time when you were not at school or work?^a^	36 (17.7)	3.92	1.34
Physical neglect			
Did your parents/guardians not give you enough food even when they could easily have done so?	62 (30.5)	2.56	1.14
Were your parents/guardians too drunk or intoxicated by drugs to take care of you?	18 (8.9)	2.08	0.62
Did your parents/guardians not send you to school even when it was available?	25 (12.3)	2.12	0.72
ACE counts			
0	27 (13.3)	2.33	1.11
1	30 (14.8)	2.22	0.79
2–3	62 (30.5)	2.15	0.68
4+	84 (41.3)	2.29	1.13
ACEs exposure^b^			
Yes	176 (86.7)	–	–
No	27 (13.3)	–	–

*Note*. ^a^For this question, it’s the “*no*” answer which scores a “1”; ^b^ACEs Exposure (Addition of 1, 2–3, and 4+ ACEs); ^c^Sample, Yes responses only except emotional neglect

### Prevalence of psychological distress

More than half of the participants (54.7%) experienced psychological distress. A higher proportion of female students than male students experienced psychological distress (64.5% vs. 49.4%) (Table 4).

**Table 4:** Prevalence of Psychological Distress by Gender, May to July 2018, Nsukka, Nigeria

Response Categories	All N (%)	Gender	
		Male N (%)	Female N (%)
None of the time	30 (14.8)	19 (63.3)	11 (36.7)
A little of the time	71 (35.0)	28 (39.4)	43 (60.6)
Some of the time	87 (42.9)	33 (37.9)	54 (62.1)
Most of the time	12 (5.9)	3 (25.0)	9 (75.0)
All of the time	3 (1.4)	2 (66.7)	1 (33.3)
Dichotomized PD scores			
Absence	92 (45.3)	43 (56.6)^a^	49 (53.5)^a^
Presence	111 (54.7)	42 (49.4)^a^	69 (64.5)^a^

*Note*. Absence of psychological distress = K10 < 20, Presence of psychological distress = K10 ≥ 20

^a^ Within Gender

### Associations between ACEs, psychological distress, and sociodemographic characteristics

Furthermore, gender was significantly associated with ACE exposure (*p* = 0.048). Parental education was significantly associated with both ACEs exposure (*p* = 0.038) and psychological distress (*p* = 0.027) (Table 5). Additionally, parental income was significantly associated with students’ psychological distress (*p* = 0.040). We included these variables in the binary logistic and multivariable logistic regressions.

**Table 5:** Associations between adverse childhood experiences, psychological distress, and sociodemographic characteristics, May to July 2018, Nsukka, Nigeria

	ACEs exposure			Psychological distress		
	Yes	No		Yes	No	
	(*n* = 167)	(*n* = 36)	*p-value	(*n* = 111)	(*n* = 92)	*p-value
	*n* (%)	*n* (%)		*n* (%)	*n* (%)	
Age						
18–20 years	90 (53.9)	22 (61.1)	0.442	62 (55.9)	50 (54.3)	0.347
21–24 years	50 (29.9)	7 (19.4)		34 (30.6)	23 (25.0)	
≥ 25 years	27 (16.2)	7 (19.4)		15 (13.5)	19 (20.7)	
Gender						
Male	63 (37.7)	20 (55.6)	0.048	42 (37.8)	41 (44.6)	0.332
Female	104 (62.3)	16 (44.4)		69 (62.2)	51 (55.4)	
Academic level						
100 Level	49 (29.3)	10 (27.8)	0.958	26 (23.4)	33 (35.9)	0.210
200 Level	41 (24.6)	9 (25.0)		31 (27.9)	19 (20.7)	
300 Level	54 (32.3)	13 (36.1)		40 (12.6)	17 (14.1)	
400 Level	23 (13.8)	4 (11.1)		14 (12.6)	13 (14.1)	
Birth order						
1st child	44 (26.3)	10 (27.8)	0.934	28 (25.2)	26 (28.3)	0.156
2nd child	44 (26.3)	9 (25.0)		31 (27.9)	22 (23.9)	
3rd child	14 (13.2)	2 (5.60)		13 (11.7)	3 (3.3)	
≥ 4th child	32 (19.8)	6 (16.7)		17 (15.3)	21 (22.8)	
Last child	33 (19.8)	9 (25.0)		22 (19.8)	20 (21.7)	
Parental education						
No formal education	39 (23.4)	6 (16.7)	0.038	31 (27.9)	14 (15.2)	0.027
Primary education	31 (18.6)	6 (16.6)		24 (21.6)	13 (14.1)	
Secondary education	68 (40.7)	10 (27.8)		38 (34.2)	40 (43.5)	
Tertiary education	29 (17.4)	14 (38.9)		18 (16.2)	25 (27.2)	
Parental income						
Low income	63 (37.7)	14 (38.9)	0.975	39 (35.1)	38 (41.3)	0.040
Middle	64 (38.3)	14 (38.9)		51 (45.9)	27 (29.3)	
High income	40 (24.0)	8 (22.2)		21 (18.9)	27 (29.3)	

*Note*. *p-values for the chi-square test

The logistic regression analysis showed that sexual abuse (OR = 2.36; 95% CI: 2.36, 7.65), physical neglect (OR = 2.87; 95% CI: 1.57, 5.31), ACE exposure, i.e., exposure to any form of adversities (OR = 6.66; 95% CI: 2.41, 18.42), having 1 ACE (OR = 4.40; 95% CI: 1.32, 14.70), having 2–3 ACEs (OR = 4.13; 95% CI: 1.39, 12.29), and having 4+ (OR = 11.67; 95% CI: 3.95, 34.45) were significantly associated with psychological distress (Table 6). The results of the multivariable logistic regressions showed that the participants whose parents had secondary education (AOR = 0.35; 95% CI: 0.16, 0.79) and tertiary education (AOR = 0.30; 95% CI: 0.12, 0.74), respectively were 0.35 times and 0.30 times less likely than the participants whose parents had NFE to experience psychological distress. Also, the odds of experiencing psychological distress was 2 times (AOR = 2.19; 95% CI: 1.11, 4.32) higher among the students of parents with middle income level. Regarding ACEs exposure, we observed non-significant associations for gender, parental education, and parental income among the participants. Nevertheless, the odds of experiencing ACEs exposure was 2.21 times (AOR = 2.21; 95% CI: 0.95, 5.11) higher among male students than the female students (Table 7).

**Table 6:** Association between adverse childhood experiences and psychological distress among the participants May to July 2018, Nsukka, Nigeria

	Psychological distress					
	Presence	ORAbsence				
	*n*(%)	*n*(%)	95% CI	*P-*value		
ACEs						
^a^Household dysfunction						
No	72 (52.2)	66 (47.8)	1.38	0.76	2.50	0.297
Yes	39 (60.0)	26 (40.0)				
^b^Sexual abuse						
No	34 (36.2)	60 (63.8)	4.25	2.36	7.65	< 0.0001
Yes	77 (70.6)	32 (29.4)				
^c^Emotional abuse						
No	26 (44.1)	33 (55.9)	1.83	0.99	3.37	0.053
Yes	85 (59.0)	59 (41.0)				
^d^Physical abuse						
No	24 (48.0)	26 (52.0)	1.43	0.75	2.71	0.276
Yes	87 (56.9)	66 (43.1)				
^e^Emotional neglect						
No	106 (55.2)	86 (44.8)	0.68	0.20	2.29	0.530
Yes	5 (45.5)	6 (54.5)				
^f^Physical neglect						
No	60 (45.8)	71 (54.2)	2.87	1.57	5.31	0.001
Yes	51 (70.8)	21 (29.2)				
^g^ACEs Exposure						
No	5 (18.5)	22 (81.5)	6.66	2.41	18.42	< 0.0001
Yes	106 (60.2)	70 (39.8)				
ACE count						
0 (ref)	5 (18.5)	22 (81.5)				
1	15 (50.0)	15 (50.0)	4.40	1.32	14.70	0.016
2–3	30 (48.4)	32 (51.6)	4.13	1.39	12.29	0.011
4+	61 (72.6)	23 (27.4)	11.67	3.95	34.47	< 0.0001

*Note*. *ACEs* adverse childhood experiences, *OR* odds ratio; *95% CI* 95% confidence intervals

^a^No is the reference category

^b^No is the reference category

^c^No is the reference category

^d^No is the reference category

^e^No, reference category

^f^No, reference category

^g^No/Absence of any adverse events, reference category

***p-value < 0.0001. **p-value < 0.01. *p-value < 0.05

**Table 7:** Association between adverse childhood experiences exposure, psychological distress by demographic characteristics of students May to July 2018, Nsukka, Nigeria

Variables	ACEs Exposure				Psychological distress			
	Yes	No			Presence	Absence		
	*n*(%)	*n*(%)	AOR (95% CI)	*P-value*	*n*(%)	*n*(%)	AOR (95%CI)	*P-value*
Gender								
Female (ref)	109 (90.8)	11 (9.2)			69 (57.5)	51 (42.5)	1	
Male	67 (80.7)	16 (19.3)	2.21 (0.95, 5.11)	0.065	42 (50.6)	41 (49.4)	1.36 (0.75, 2.46)	0.317
Parental Education								
NFE (ref)	39 (86.7)	6 (13.3)	1		31 (68.9)	14 (31.1)	1	
PRY EDU	34 (91.9)	3 (8.1)	1.56 (0.35, 6.86)	0.560	24 (64.9)	13 (35.1)	0.79 (0.30, 2.04)	0.619
SEC EDU	69 (88.5)	9 (11.5)	1.08 (0.35, 3.34)	0.892	38 (48.7)	40 (51.3)	0.35 (0.16, 0.79)	0.012
TER EDU	34 (79.1)	9 (20.9)	0.57 (0.18, 1.80)	0.341	18 (41.9)	25 (58.1)	0.30 (0.12, 0.74)	0.009
Parental income								
Low income (ref)	66 (85.7)	11 (14.3)	1		39 (50.6)	38 (49.4)	1	
Middle income	67 (85.9)	11 (14.1)	1.07 (0.42, 2.72)	0.886	51 (65.4)	27 (34.6)	2.19 (1.11, 4.32)	0.024
High income	43 (89.6)	5 (10.4)	1.55 (0.49, 4.88)	0.453	21 (43.8)	27 (56.3)	0.81 (0.38, 1.71)	0.575

*Note*. *AOR* adjusted odds ratio, *95% CI* 95% confidence intervals, *ref.* reference category; Hosmer and Lemeshow test, *p*-value = 0.13 (model 1); Hosmer and Lemeshow test, *p*-value = 0.874 (model 2)

**p-value < 0.001. *p-value < 0.05

## Discussion

This study offered more evidence on the prevalence of ACEs, psychological distress, and the relationship between these outcomes among higher education students. Overall, our finding suggests that many participants experienced at least one form of ACEs before 18 years. The prevalence of one or more ACEs is higher than those reported in prior studies [14, 53–57]. The high proportion of students who experienced ACEs in this study reflects the family or neighborhood conditions many Nigerian children are growing up, and young people live. Consistent with previous Nigerian studies [25, 26], this finding confirmed our first hypothesis. The high prevalence of ACEs in this study portends danger for future adult health. Since self-reported health is an established strong predictor of morbidity and mortality in adults [31, 58], young people might have experienced more adversities during childhood or adolescence. Therefore, our finding provides ample opportunity for quality assessment of conditions in which children are raised and growing up in Nigeria to modify these conditions and ameliorate such adversities through family, school-and community-based mental health interventions. Furthermore, there is a need to improve the social, structural, economic, and cultural conditions where children are raised and mitigate factors that undermine positive health outcomes among young adults, especially for girls and young female adolescents in Nigeria since evidence suggests that poor conditions contribute to the risk of exposure to childhood adversities [59, 60]. Also, factors that promote ACEs such as inequality, gender-based violence, low level of schooling should be addressed with available resources, political will, and government interventions in terms of youth empowerment programs and free and quality education. Since low-income family environments enhance childhood adversity with adverse health outcomes, opportunities in education, employment, and active participation in both political and economic sectors for young people can help mitigate ACEs. Consistent with prior studies’ findings, the high prevalence of ACEs has been reported among young adult populations [25, 30, 59, 60].

Additionally, a considerable proportion of the participants reported experiencing 1 ACE, 2–3 ACEs, and 4+ ACEs. The experience of different categories of childhood adversity varied in our sample. The variations in ACEs exposure may be attributed to diverse family factors and neighborhood conditions (e.g., living in poor homes, exposure to family, and communal violence) in which the students grew up. Remarkably, about one-fourth of our sample reported experiencing 4+ ACEs. This proportion is higher than the findings reported in previous studies [31, 32, 59, 60]. The students with ≥2 ACEs are more likely to be eligible for special healthcare needs than those with no ACE. Identification of students with multiple ACEs via school, health facility, and community-based mental health screenings can provide anticipatory guidance strategies more relevant and responsive to youth and family context [32]. Furthermore, there is a need for collective efforts to change the existing system that perpetuates risk factors for ACEs across every sphere of life in Nigeria. For instance, the Child’s Rights Act enacted in Nigeria in 2003 does not guarantee or protect Nigerian children’s rights due to lack of political will and ineffective implementation. Thus, the rights of the child are neglected in many Nigerian households and communities. Therefore, concerted efforts aimed at implementing the Child’s Rights Act should be taken by the government and child rights advocates. Furthermore, women’s empowerment via access to quality education and economic opportunities can help mitigate or prevent ACEs among poor households.

The participants also reported that emotional abuse and physical abuse were the most frequently experienced forms of ACEs. Prolonged exposure to emotional abuse and physical abuse during childhood and adolescence is linked to chronic health conditions, emotional health problems, health risk behaviors, and impairs academic/educational attainment in youth [2, 61]. Since exposure to emotional and physical abuse occurs during childhood and adolescence, it may have a cumulative effect on the quality of life, life expectancy in adulthood and increase the risk for future chronic diseases [57, 62]. This finding is consistent with prior studies [31, 32]. Further studies using longitudinal designs should explore factors associated with a high prevalence of emotional and physical abuse among young Nigerian adults.

Similarly, more than half of the participants experienced psychological distress. There is much evidence that ACEs are potential predictors of psychological distress in young people [1, 2, 4, 7, 24, 56, 63]. If unmitigated, psychological distress might lead to poor educational attainment, poor mental health outcomes, and youth unemployment by adversely affecting job performance and increasing absenteeism in later adulthood [2, 61, 63, 64]. The finding validates the socioecological model’s tenets that posit that many factors interact to influence health outcomes, including psychological outcomes. However, modifying the susceptibility factors/conditions (a lack of family bond, poverty, violent neighborhoods, poor school-connectedness, and school climate) that foster ACEs and later PD in young adults have been shown to produce positive outcomes under supportive context [65, 66].

Interestingly, the proportion of students who experienced PD in our study was higher than previously reported in the literature [67–69]. The high prevalence of PD in our sample is of great concern. Evidence suggests that PD undermines the development of full potentials and decreases job opportunities in the affected youths [66, 69]. For instance, entrepreneurs/employers may discriminate against job seekers with potential mental health problems, particularly in Nigerian labor markets, with limited opportunities. Also, there is evidence that young people with mental health problems engage in substance use [64, 66, 69] (e.g., substance use and abuse are highly prevalent among Nigerian youths), such risky behavior is associated with adverse health outcomes in young adults. Therefore, it becomes imperative to implement evidence-based interventions that integrate capacity-building initiatives and support systems for young adults with mental health problems by community health workers, health education experts, NGOs, and governmental health agencies at the family, school, and community levels to assist this high-risk group. The interventions may enable this category of youth to adjust to life better. The finding is consistent with prior studies [66–69].

In our study, female students reported more psychological distress than their male counterparts. It may be that female students’ higher level of exposure to ACEs probably contribute significantly to their experience of psychological distress. The finding should be considered in terms of cultural practices (girl-child neglect, son preference, early marriage) that predispose young female adults to poor psychological health outcomes. This finding is consistent with previous studies [5, 11, 14, 30, 56, 67, 70]. Therefore, gender-specific psychological health interventions should be designed and implemented to identify female students with psychological distress for adequate and prompt care. Future studies should explore gender differences in childhood adversity exposure and develop poor psychological outcomes via longitudinal/cohort studies. Such studies may provide us with valuable information and an apt understanding of gender differentials in ACEs and psychological distress in later adult life in the Nigerian context.

The correlational analysis showed a positive relationship between ACEs and PD in our sample. This finding confirms earlier findings that childhood adversity is strongly linked with poor mental health outcomes in youth [3–5, 30]. This finding provides viable opportunities for developing prevention and intervention strategies about providing youth-friendly mental health services with multifaceted approaches (clinical approaches, community-based mental health screening, preventive care) targeted at identifying young adults with ACEs and their associated outcomes. The interventions may improve the overall health and well-being of teeming Nigerian youth.

In the bivariate analysis, gender was significantly associated with ACE exposure, and parental education was significantly associated with both ACEs exposure and psychological distress. Further, parental income was significantly related to only psychological distress. Research has shown that a mixture of risk factors has been linked to ACEs and psychological consequences in childhood, adolescence, and later adulthood. For instance, Sidebotham et al. [71] and Thornberry et al. [72] reported that these risk factors manifest across diverse spheres such as parental background, individual, family environment, and socioeconomic context. This finding agrees with previous studies [5, 11, 14, 67, 73]. These findings indicate the need to identify and further explore the roles of multiple risk factors for childhood adversities and their direct links to poor psychological health outcomes in the youth population with more emphasis on developing nations because the clustering of multiple risk factors increases the likelihood of ACEs [72, 73]. Also, evidence-based interventions to increase family income and improve parental education are necessary for Nigeria at the policy-making level. There would be a need for an inclusive, more substantial, and macro-economic change in the country [74]. A large-scale job creation program for adults in Nigeria could economically empower many parents and lift them out of poverty. Since access to quality education can improve the population, government and NGOs need to provide affordable and quality education for teeming Nigerian youths who are future parents. These interventions may drastically reduce the risks of childhood adversities among the Nigerian population because research indicates that higher socioeconomic status promotes population health [74, 75].

The logistic regression analysis showed that sexual abuse, physical neglect, ACE exposure (i.e., exposure to any form of adversities), having at least 1 ACE, 2–3 ACEs, and 4+ were significantly associated with psychological distress in our sample. Sexual abuse is a potential risk factor for poor mental outcomes in young people, and children are more likely than adolescents to be victims of sexual abuse [76, 77]. Furthermore, adolescents suffer exposure to the most intrusive sexual offenses than children and are more likely to experience sexual violence with poor mental health outcomes [76]. This finding may offer a potential explanation for the observed association between sexual abuse and later onset of PD in our subjects. The result points to the urgent need for sexual abuse prevention programs (comprehensive sexuality education) by health educators or sexual health practitioners in the university environment and providing appropriate support services to the victims of sexual abuse during childhood or adolescence. The finding also has implications for education programs that increase the sexual abuse awareness of relevant professionals, use of appropriate actions when professionals recognize such cases, and thus increase the possibility that adolescents and youth would receive potential mental, health, and legal support [76].

Furthermore, systematic screening with appropriate clinical interventions at the health facilities could alleviate the outcomes of sexual abuse [31]. Further, higher ACE counts were also associated with PD in our sample. Young people with 4+ ACEs are more likely to be unemployed, suffer chronic illnesses, and drop-out of school [30]. Such findings suggest comprehensive and elaborate preventive care, such as mental screenings for young people via the school and healthcare settings. Future work using larger samples should explore if other forms of ACEs are associated with psychological outcomes in young adults with a history of ACEs. The findings are consistent with previous studies [2, 6, 7, 10, 30–32].

Furthermore, the multivariable logistic regression results showed that the participants whose parents had secondary education and tertiary education were 0.35 times and 0.30 times less likely than the participants whose parents had NFE to experience psychological distress. Research evidence suggests that parental education provides buffers against poor health outcomes among children and adolescents [10, 57]. The plausible explanation could be that parents with secondary and tertiary education can adopt effective protection measures for ACEs, especially in the family, neighborhoods, and schools. The finding is consistent with a previous study [57, 78] that reported parental education as a protective factor against ACEs. Thus, it may be particularly crucial for the government and other stakeholders to implement measures that facilitate access to quality education by the parents. This intervention, hopefully, may reduce the prevalence of ACEs for all children in Nigeria. Future studies should explore other protective factors such as strong supporting family relationships (i.e., non-violent parents and older adults), safe student-teacher relationship, peer relationship, neighborhood collective efficacy, and school connectedness with the potential to mediate or moderate the impact of ACEs on psychological outcomes in early and later adulthood [57, 78].

Also, the odds of experiencing psychological distress was 2 times higher among the students of parents who are middle-income earners. Thus, improving families’ economic conditions and providing access to quality education for low SES people in Nigeria may present a vital tool for reducing the prevalence of childhood adversity. This finding has implications for interventions that focus on improving family contexts (family environments, household characteristics, household income, relationships between adolescents and household members) and those targeted at ameliorating childhood adversity and associated outcomes in adolescent and youth populations. We observed non-significant associations for gender, parental education, and parental income among the participants regarding ACEs exposure. Nevertheless, the odds of ACEs exposure were higher among male students than female students.

### Strength and limitations

While this study has provided notable findings on the prevalence of ACEs, psychological distress, and association with some demographic factors in young Nigerian adults, it has several limitations. Thus, caution should be taken when considering our findings. One of the study limitations is that the data are cross-sectional; this study’s cross-sectional design limits the ability to infer causation concerning the associations between ACEs and psychological distress. The study was conducted among students in higher educational settings; therefore, the findings may not be generalizable to youth not enrolled in any higher institutions. Another limitation of the study is that our sample was made up of higher education students. This may suggest that the participants have higher socioeconomic status (SES) compared to those who do not have access to higher education or youths with low SES. Since access to higher education in Nigeria is expensive, majority of our sample may be from high income households with little experience of ACEs. Thus, we are unable to generalize the findings to young adults with low SES or from low-income households. Furthermore, we had a considerable proportion of attrition in this study. This situation may be attributed to students’ reluctance to give information on their adverse childhood experiences, which is considered a sensitive issue that should only be restricted to the family in many Nigerian communities. Therefore, future studies should examine ACEs among young adults using longitudinal and mixed methods designs. Another limitation was the use of self-reported questionnaires, which could introduce recall and response bias. For instance, the study participants could underestimate or overestimate some information since ACEs were reported during adulthood. Also, it would have been helpful to have examined PD with a clinical diagnosis. However, limited resources at our disposal and the relatively large sample size made this approach infeasible. To mitigate these inherent limitations, the researchers underwent extensive training to standardize the participants’ instructions during the interview process. Also, we used well-validated instruments for data collection.

Further, there is evidence that youth self-report of ACEs is reliable and valid in other environments [79]. Despite these limitations, our research has significant implications for adolescents’ and young adults’ health, especially the primary prevention of ACEs and poor health outcomes. Future studies are recommended to examine the prevalence of ACEs, their association with other health outcomes, and the roles of the familial, neighborhood, and school protective factors for youth with ACEs through longitudinal population-based designs focusing on developing countries.

## Conclusions

ACEs are prevalent among young adults and are associated with psychological distress in adulthood. Furthermore, parental factors are associated with ACEs and psychological distress. Thus, implementation of school, community-and facility-based routine mental health screening programs is essential for prompt identification, prevention, and treatment of youth with childhood adversities and poor mental health outcomes. Besides, routine ACEs screening should be integrated into adolescent and youth preventive care in primary health care and higher educational settings. Also, the findings underscore the need for public health education interventions and advocacy programs that focus on preventing and eliminating the risk factors for ACEs in the communities. For instance, the community-based health education programs should be targeted at household heads/parents, traditional, political, and religious leaders who can help change established cultural norms that foster ACEs. The parents and leaders should be informed about the childhood and long-term adverse health outcomes of ACEs on the individuals and preventive measures for ACEs at the household, school and community levels. Also, advocacy programs by public health experts, health education teachers, social workers and child rights activists could facilitate the enforcement of the Child Rights Acts in Nigeria. Effective implementation of the Child Rights Acts can prevent ACEs in Nigerian communities. Similarly, efforts should be made to provide safe and healthy environments for all Nigerian children at the family, school, and community levels. The measures may enhance the prevention and mitigation of childhood adversities among adolescents and youth in Nigeria.

## Supplementary Information

**Additional file 1.**

**Additional file 2.**

**Additional file 3.**

## Data Availability

The dataset(s) supporting the conclusions of this article is (are) included within the article (and its additional file(s).

## Abbreviations

CI: Confidence interval
ACE: Adverse childhood experience
ACE-IQ: Adverse Childhood Experiences International Questionnaire
PD: psychological distress
SES: Socioeconomic status
VACS: Violence Against Children Survey
VIF: Variance Inflation Factor
AOR: Adjusted odds ratio
OR: Odds ratios
LGA: Local Government Area
NFE: No Formal Education
NGOs: Non-governmental organizations
