# Correction to: Adverse childhood experiences and psychological distress among higher education students in Southeast Nigeria: an institutional-based cross-sectional study

**DOI:** 10.1186/s13690-021-00625-0

**Published:** 2021-06-10

**Authors:** Olaoluwa Samson Agbaje, Chinwe Patience Nnaji, Evelyn Nwanebe Nwagu, Cylia Nkechi Iweama, Prince Christian Ifeanachor Umoke, Lawretta Eyuche Ozoemena, Charles Chike Abba

**Affiliations:** Department of Human Kinetics and Health Education, Faculty of Education, University of Nigeria, Nsukka, Enugu State Nigeria

**Correction to: Arch Public Health 79, 62 (2021)**

**https://doi.org/10.1186/s13690-021-00587-3**

Following publication of the original article [1], the authors reported an error in the 3rd author’s middle name, which was wrongly spelled as "Nwanabe”.

The correct spelling of the middle name is "Nwanebe."

The author name has been revised in this Correction and the original article [1] has been updated as well.
