# Supplementary material for: Adverse childhood experiences and psychological distress among higher education students in Southeast Nigeria: an institutional-based cross-sectional study
Source: Arch Public Health. 2021 Apr 29;79:62. doi: 10.1186/s13690-021-00587-3 (PMC8086118; doi:10.1186/s13690-021-00587-3)
Supplement: Supplementary file 1 — Additional file 1. [file 13690_2021_587_MOESM1_ESM.docx]

Distribution plots for power analysis

Protocol of power analysis for *X* and *Y* plot for a range of values

[1] *-- Saturday, May 25, 2019 -- 16:43:39*

**z tests -** Logistic regression

**Options:** Large sample z-Test, Demidenko (2007) with var corr

**Analysis:** Post hoc: Compute achieved power

**Input:** Tail(s) = Two

Odds ratio = 2.3333333

Pr(Y=1|X=1) H0 = 0.3

α err prob = 0.05

Total sample size = 330

R² other X = .1

X distribution = Normal

X parm μ = 0

X parm σ = 1

**Output:** Critical z = 1.9599640

Power (1-β err prob) = 0.9999957
